# Nodal staging in rectal cancer: why is restaging after chemoradiation more accurate than primary nodal staging?

**DOI:** 10.1007/s00384-016-2576-8

**Published:** 2016-04-07

**Authors:** Luc A. Heijnen, Monique Maas, Regina G. Beets-Tan, Myrthe Berkhof, Doenja M. Lambregts, Patty J. Nelemans, Robert Riedl, Geerard L. Beets

**Affiliations:** Department of Radiology, Maastricht University Medical Center, Maastricht University, PO Box 5800, 6202 Maastricht, The Netherlands; Department of Surgery, Maastricht University Medical Center, Maastricht University, PO Box 5800, 6202 Maastricht, The Netherlands; Department of Radiology, The Netherlands Cancer Institute, Amsterdam, The Netherlands; GROW-School for Oncology and Developmental Biology, PO Box 616, 6200 Maastricht, The Netherlands; Department of Epidemiology, Maastricht University Medical Center, Maastricht University, PO Box 5800, 6202 AZ Maastricht, The Netherlands; Department of Pathology, Zuyderland Medical Centre, PO Box 5500, 6130 MB Sittard, The Netherlands; Department of Surgery, The Netherlands Cancer Institute, Amsterdam, The Netherlands

**Keywords:** Rectal cancer, Nodal staging, Chemoradiation, Response, Histopathology

## Abstract

**Purpose:**

This study aims to explore the influence of chemoradiation treatment (CRT) on rectal cancer nodes and to generate hypotheses why nodal restaging post-CRT is more accurate than at primary staging.

**Methods:**

Thirty-nine patients with locally advanced rectal cancer underwent MRI pre- and post-CRT. All visible mesorectal nodes were measured on a 3D T1-weighted gradient echo (3D T1W GRE) sequence with 1-mm^3^ voxels and matched between pre- and post-CRT-MRI and with histology by lesion-by-lesion matching. Change in number and size of nodes was compared between pre- and post-CRT-MRI. ROC curves were constructed to assess diagnostic performance of size.

**Results:**

Eight hundred ninety-five nodes were found pre-CRT: 44 % disappeared and 40 % became smaller post-CRT. Disappearing nodes were initially significantly smaller than nodes that remained visible post-CRT: 2.9 mm vs. 3.8 mm. cN+ stage was predicted in 97 % pre-CRT and 36 % of patients had ypN+ post-CRT. ypN+ patients had significantly larger nodes than ypN0 patients both pre- and post-CRT. Optimal size cutoff for post-CRT ypN stage prediction was 2.5 mm (area under the curve (AUC) of 0.78) at MRI.

**Conclusions:**

After CRT, most lymph nodes become smaller, and many disappear. Size predicts disappearance and node positivity. Together with a low prevalence of ypN+, this can explain the higher accuracy of nodal staging after CRT than in a primary staging setting, possibly of use when considering organ-preserving strategies after CRT.

## Introduction

Multimodality neo-adjuvant chemoradiation treatment (CRT) for patients with locally advanced rectal cancer patients leads to significant changes in the number and distribution of rectal cancer lymph nodes in the mesorectum [1–5]. These changes can have an impact on the accuracy of the radiological and histological nodal staging after surgery. Recent studies have reported that radiological nodal staging after CRT is more sensitive than at primary staging imaging, with negative predictive values of up to 95 % for prediction of a ypN0 status on restaging MRI [6, 7]. Several studies have also suggested a reduction in nodal number and size after CRT as a reason for a low nodal harvest at histological examination after CRT [8, 9]. Accurate nodal restaging is becoming clinically more important when an organ-preserving treatment, rather than a total mesorectal excision, is considered after a good response to CRT.

The aim of this study was to explore the influence of CRT on the number and size of lymph nodes, and to generate hypotheses why nodal restaging on post-CRT MRI is more accurate than at primary nodal staging MRI.

## Material and methods

### MR imaging

Included in this study were consecutive patients with locally advanced rectal cancer, defined as (1) T4 or T3 tumour with a threatened or involved mesorectal fascia, (2) a distally located T3N1 tumour or (3) N2-status. MRI criteria for nodal involvement at primary staging were a size larger than 5 mm, an irregular border or shape and/or a heterogeneous signal of the node [10]. Patients provided written informed consent for the use of their data as part of a larger study on nodal staging with MRI [11]. All patients underwent MRI (before and 6–8 weeks after CRT) at a 1.5 T MR unit (Intera; Philips Medical Systems, Best, The Netherlands) with a phased-array body coil. Patients did not receive bowel preparation or spasmolytics. Sequences were standard 2D T2-weighted fast spin echo (2D T2W FSE) sequences (TR/TE 3427/150 msec, 90° flip angle, 25 echotrain length, 3–5-mm-slice thickness, 2 mm gap, 6 NSA, 175 × 256 Matrix, 200 FOV, 308-s acquisition time) in three planes (sagittal, axial and coronal) and an additional axial 3D T1-weighted gradient echo (3D T1W GRE) sequence (TR/TE 9.8/4.6 msec, 15° flip angle, 1-mm-slice thickness, 1 NSA, 384 × 384 Matrix, 440 mm FOV, 391-s acquisition time). The axial sequences were angulated perpendicular to the tumour axis. Total scan time was 42 min. All patients underwent neo-adjuvant CRT and the aforementioned MRI protocol was repeated 6–8 weeks after the last radiation treatment to evaluate the response to CRT.

### Image evaluation

**Fig. 1:**
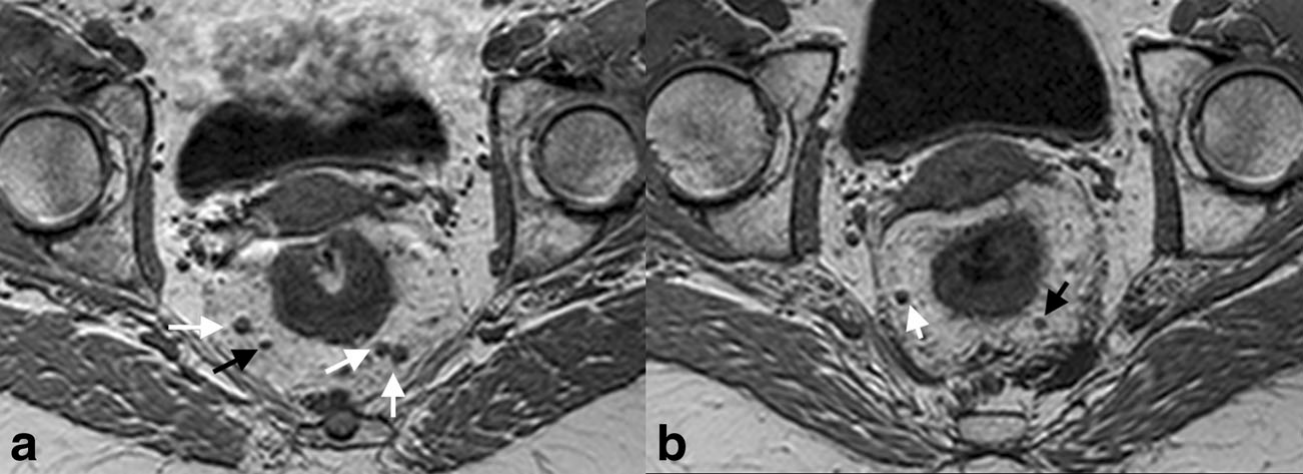
3D T1-weighted gradient echo images before (**a**) and after (**b**) CRT. The pre-CRT image shows four lymph nodes (*arrows*). The two nodes on the *left* and the lateral node on the *right* (*white arrows*) are just over 5 mm in size and the two nodes on the left side also show irregular borders. These nodes were therefore interpreted as malignant nodes, while the smaller node (*black arrow*) was interpreted as benign. After CRT (**b**), two nodes are no longer visible and are assumed to be benign, whereas the two remaining nodes have decreased in size. The two remaining nodes are 2–3 mm (*black arrow*) and 4 mm (*white arrow*) in size, without suspicious characteristics. They were interpreted as benign nodes, confirmed at histopathology

The images were evaluated by a radiologist with 4 years of experience in reading rectal MRI (150 scans per year). Each node in the mesorectum that was visible on the pre-CRT MRI was matched with its equal on the post-CRT MRI. For each node, the size (short axis diameter) was measured on the 3D T1W GRE sequence (Fig. 1). If a node had disappeared on the post-CRT MRI, this was noted. The nodes were drawn on an anatomical map to ensure accurate lesion-by-lesion matching between the pre-CRT, post-CRT images and histology. Apart from the size and location, T-stage, N-stage and distance from tumour to the tumour to the mesorectal fascia were evaluated with T2W-MRI according to earlier reported criteria [12, 13].

### Treatment and histology

Neo-adjuvant CRT consisted of 28 fractions of 1.8 Gy on weekdays with capecitabine 825 mg/m^2^ twice daily during radiation on both week and weekend days. Surgery consisted of total mesorectal excision as described by Heald et al. [14]. Histological examination was performed according to standard methods described by Quirke et al. [15] by a pathologist subspecialized in gastro-intestinal pathology. Post-CRT imaging findings were correlated with the histological nodal findings by lesion-by-lesion matching. This methodology was described in detail in previous reports on nodal imaging with lesion-by-lesion validation [11]. The surgical resection specimen was sectioned perpendicular to the rectal lumen to match with the MR images. Lesion-by-lesion nodal matching was obtained by side-by-side comparison of the anatomical map with the axially sliced specimen. T-downstaging was defined as a decrease between T-stage at pre-CRT imaging and final T-stage at histology. Nodal downstaging was defined as a decrease in N-stage between pre-CRT MRI and final N-stage at histopathology.

### Statistical analyses

Baseline characteristics were collected, including several patient and tumour-related factors. Descriptive statistics were used to evaluate node characteristics. For analysis purposes, we assumed that nodes that disappeared after CRT were benign or sterilised. To investigate our aim, we compared the nodal number and size before and after CRT and between patients with and without involved nodes at histology. Baseline characteristics were compared between patients with and without involved nodes at histology as well. The independent samples *T* test was used for continuous variables and the *χ*^2^ test was used to compare proportions. For comparisons of repeated measurements, the paired samples *T* test was used. Statistical analyses were performed with SPSS version 16.0 and Stata version 11.0.

## Results

### Baseline characteristics

In total, 39 patients with locally advanced rectal cancer were included. Median age was 70 (38–87) years. At baseline, the MRI was suggestive of nodal involvement in 38 of 39 patients (97 % cN+). The mean interval between the last radiation fraction and the post-CRT MRI was 5.8 (±SD 1.8) weeks. In total, 44/453 nodes were malignant at histopathology leading to a ypN+ status in 14/39 patients. Baseline characteristics and details on number and size of the nodes before and after CRT are shown in Table 1, for all patients and categorised according to ypN status.

**Table 1:** Baseline characteristics and results with regard to number and size of nodes for all patients and for patients with and without positive nodes at pathology

	All (*n* = 39)	ypN0 (*n* = 25)	ypN+ (*n* = 14)	*p* value
Age (months)	70 (38–87)	70 (52–87)	65 (38–80)	*p* = 0.101
Gender (% male)	27 (69 %)	18 (72 %)	9 (64 %)	*p* = 0.617
Clinical T-stage				
cT1	0 %	0	0	
cT2	4 (10 %)	2 (8 %)	2 (14 %)	
cT3	33 (85 %)	21 (84 %)	12 (86 %)	*p* = 0.480
cT4	2 (5 %)	2 (8 %)	0	
Clinical N-stage				
cN0	1 (3 %)	1 (4 %)	0	
cN1	10 (26 %)	6 (24 %)	4 (29 %)	*p* = 0.782
cN2	28 (72 %)	18 (72 %)	10 (71 %)	
Post-CRT clinical T-stage				
yT1	0 %	0 %	0	
yT2	11 (28 %)	7 (28 %)	4 (29 %)	*p* = 0.550
yT3	26 (67 %)	16 (64 %)	10 (71 %)	
yT4	2 (5 %)	2 (8 %)	0	
Post-CRT clinical N-stage				
yN0	18 (46 %)	15 (60 %)	3 (21 %)	
yN1	14 (36 %)	7 (28 %)	7 (50 %)	*p* = 0.065
yN2	7 (18 %)	3 (12 %)	4 (29 %)	
ypT stage				
ypT0	3 (8 %)	2 (8 %)	1 (7 %)	
ypT1	4 (10 %)	4 (16 %)	0	
ypT2	10 (26 %)	7 (28 %)	3 (21 %)	*p* = 0.378
ypT3	21 (54 %)	11 (44 %)	10 (71 %)	
ypT4	1 (3 %)	1 (4 %)	0	
ypN stage				
ypN0	25 (64 %)			
ypN1	9 (23 %)	NA	NA	
ypN2	5 (13 %)			
Pre-CRT size (mean ± sd)	3.8 (±2.0)	3.6 (±1.6)	6.3 (±3.5)	*p* < 0.0001
Post-CRT size mean ± sd)	2.5 (±1.7)	2.3 (±1.4)	4.5 (±2.8))	*p* < 0.0001
Number of nodes pre-CRT (mean ± sd)	22 (±8)	24 (±13)	22 (±7)	*p* = 0.560
Number of nodes post-CRT (mean ± sd)	13 (±6)	13 (±7)	11 (±6)	*p* = 0.970
Nodal harvest at pathology	12 (±6)	10 (±6)	14 (±5)	*p* = 0.019

*NA* not applicable, *CRT* chemoradiation, *sd* standard deviation

### Nodal number and size before and after CRT

**Fig. 2:**
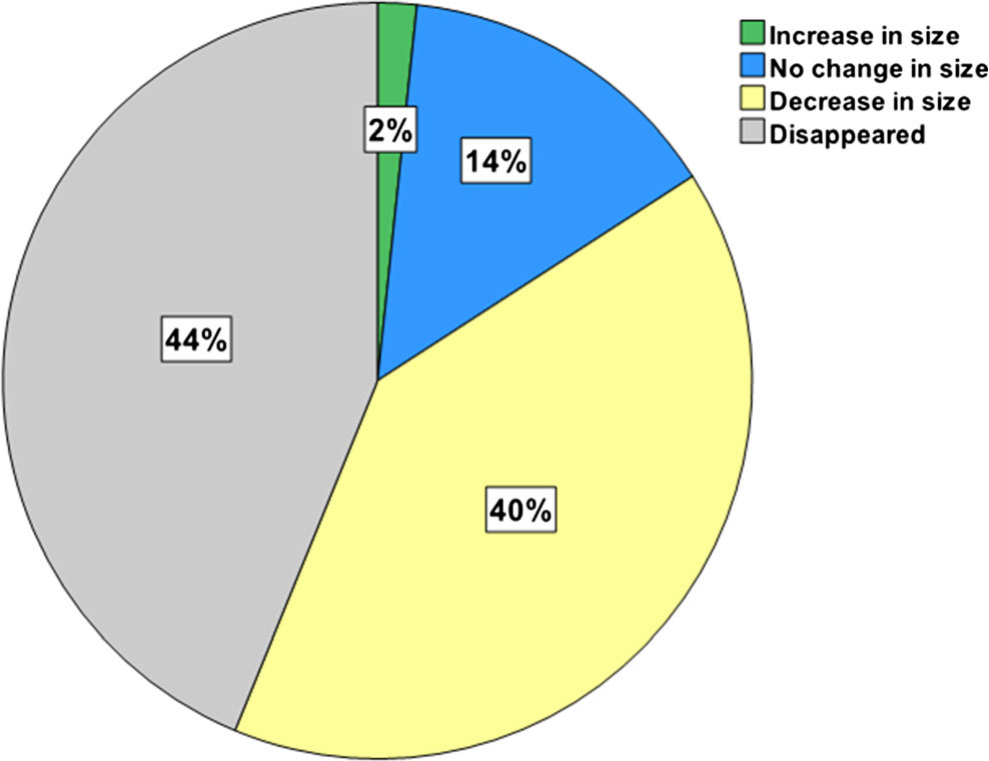
Distribution of nodal changes (in terms of size and disappearance) due to CRT

**Fig. 3:**
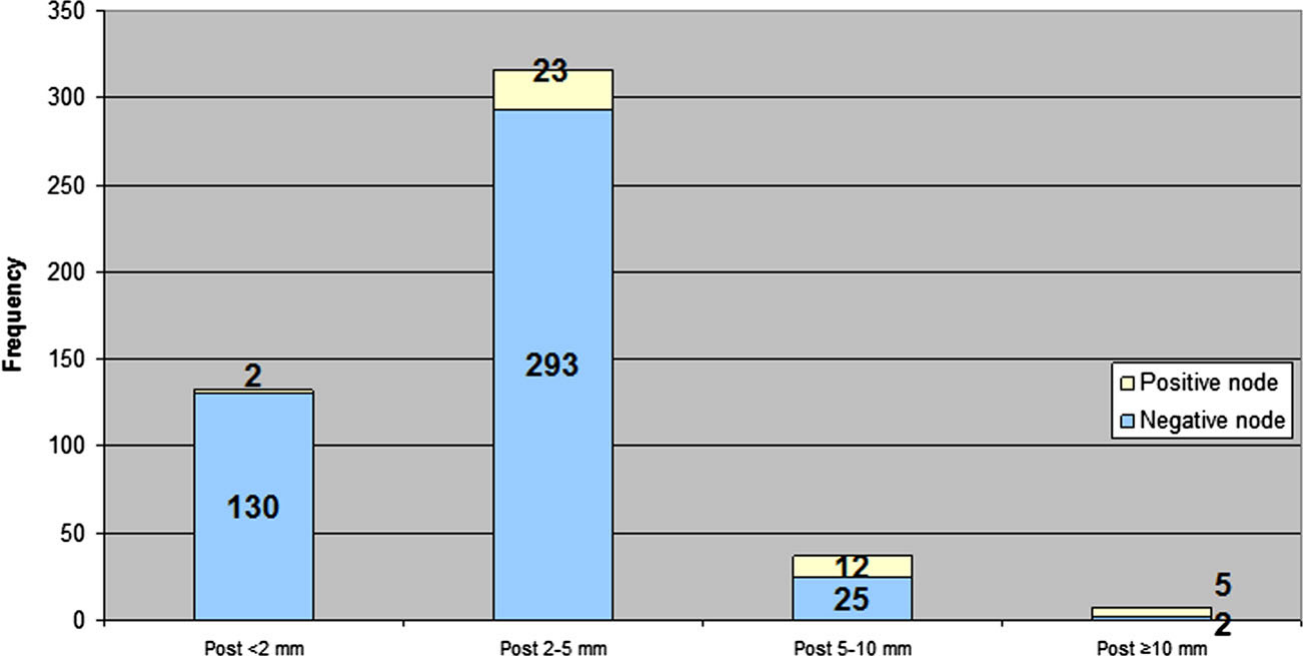
Distribution of malignant and benign nodes after CRT for different size categories

Figures 2 and 3 show changes in nodal number and size due to CRT. In the 39 patients, a total of 895 nodes were identified at pre-CRT imaging, of which 503 (56 %) could be matched with the post-CRT MR images, while 392 (44 %) were no longer visible. The 503 nodes that remained visible on MR images had a mean pre- and post-CRT size of 3.8 ± 2.0 and 2.5 ± 1.7 mm, respectively, (*p* < 0.0001). The mean pre-CRT size of the nodes that were no longer visible after CRT was 2.9 ± 1.5 mm, significantly smaller than the 3.8 ± 2.0 mm of the nodes that remained visible, (*p* < 0.0001). Of the nodes ≤5 mm at pre-CRT imaging 46 % (365/790) disappeared after CRT. Of the nodes >5 mm, a total of 26 % was not visible anymore on post-CRT MRI (27/105).

### Lymph node characteristics and prediction of nodal metastasis

Patients with positive nodal stage at histology (ypN+) had significantly larger lymph nodes, both before and after CRT, than patients with negative pathologic nodal stage (ypN0) (*p* = 0.011 before CRT and *p* = 0.036 after CRT). The total number of nodes per patient before and after CRT was not significantly different between ypN+ patients and ypN0 patients. There was no significant difference in mean number of nodes that disappeared per patient between ypN0 and ypN+ patients: 9 ± 6 vs. 11 ± 10, respectively (*p* = 0.466).

**Fig. 4:**
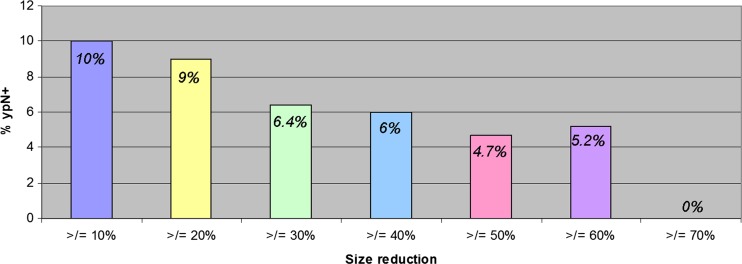
Percentage of positive nodes according to reduction in size between pre- and post-CRT MRI

After CRT size was associated with an area under curve (AUC) of 0.78 (0.71–0.86) for prediction of nodal metastasis, with optimal corresponding sensitivity and specificity of 75 and 64 %, respectively, with a 2.5 mm cutoff size. Change in size was also associated with lymph node metastasis, where a decrease in size of at least 70 % indicates ypN0 status in 100 % of the cases (Fig. 4).

## Discussion

The aim of this study was to explore the influence of CRT on the number and size of lymph nodes and to assess why nodal restaging on post-CRT MRI has a higher sensitivity and negative predictive value than in a primary staging setting. The current study shows that the majority of the nodes (84 %) become smaller (40 %) or even completely disappear (44 %) on MRI after CRT. The smaller the node, the higher the likelihood it disappears. Nodes that are not visible anymore or that substantially decrease in size at post-CRT MRI have a very low chance of harbouring metastasis. Accuracy, and mainly sensitivity, increases in the restaging setting compared to primary staging with the highest accuracy obtained at a size cutoff of 2.5 mm. This cutoff size is much lower than the generally used size of 5 mm at primary staging.

Several studies have evaluated the effect of (chemo)radiation on lymph nodes in rectal cancer. In a study by Pral et al. the biological effects of neo-adjuvant CRT on nodes in patients with rectal cancer were evaluated [16]. The authors found that irradiated nodes often show fibrous thickening of the capsule along with fibrosis or sclerosis of the nodal medulla, with a decrease in density of CD4+ T cells and dendritic cells in the nodal paracortex. In our study, the number and size of nodes is decreased, a finding that has been confirmed by other studies [3, 9, 17]. Koh et al. showed that nodes that were no longer visible after CRT had a more benign appearance before CRT than the remaining nodes, which is in line with our finding that the nodes that could no longer be identified at post-CRT MRI were significantly smaller than the nodes that remained visible [3].

Generally, AUCs of 0.72–0.75 have been reported for *primary* nodal staging in rectal cancer [11, 18, 19]. These AUCs correspond mainly with high specificities in the range of 82–88 % but with lower sensitivities of 33–58 % on a per lesion basis. In our study, a cutoff of 2.5 mm led to an AUC of 0.78 with a sensitivity of 75 % and a specificity of 64 %. This higher sensitivity after CRT with T2W-MRI has been reported previously, in the range of 80–91 % on a per lesion basis [11, 20]. The sensitivity in the current study was based on size only, while in the studies with 80–91 % sensitivity nodal staging was based on both size and morphological criteria, which is known to yield higher accuracy results [10]. The ideal cutoff of 2.5 mm in the present study is much lower than the 5 mm that has been advised for primary staging. Using the 5 mm cutoff in restaging would result in a very low sensitivity. This lower cutoff for post-CRT nodal staging has been previously identified by Lahaye et al. who found a cutoff of 3.3 mm to be optimal [7].

The current study has identified some possible mechanisms for a more accurate nodal staging with MRI after CRT as compared to primary staging at diagnosis. First, there is a tendency for the smaller nodes to disappear after CRT. Almost 50 % of the nodes ≤5 mm visible on pre-CRT MRI could not be retrieved after CRT. It is this category of small nodes in which morphology criteria are most difficult to apply. When many of these small nodes have disappeared after CRT, a radiologist will be highly confident in staging these nodes as benign after CRT, thereby improving his diagnostic performance. Additionally, the ability to compare the post-CRT with the pre-CRT MR images provides the possibility to identify changes due to CRT and thereby increases confidence in predicting the yN stage. When a node shows a remarkable decrease in size, it is highly likely that the node is benign and this increases the accuracy to select yN0 patients. Another contributing factor to the higher sensitivity of MR nodal staging in a post-CRT setting is that the prevalence of patients with malignant nodes dramatically decreases after CRT than initially predicted on MRI at primary staging: 36 vs. 97 %, respectively. This lower prevalence of positive nodes after CRT will result in a decrease in false-negative findings, further explaining the better performance in identifying a ypN0 on restaging MRI. In our study nodal response coincided with tumour downstaging in 80 % of the patients (data not shown), which is in line with other studies [3, 21, 22]. This concurrent downstaging in tumour and nodes further helps a radiologist: when there is tumour response to the CRT, he can expect to find nodal response as well.

### Clinical implications

Obtaining insight into the effect of radiation on lymph nodes is important in clinical practice. Assessment of nodal response can be helpful in the planning of the extent of the resection, i.e. when there are remaining involved nodes close to the resection margins. It is also becoming more important with the increasingly considered options of organ-preservation after a good response to CRT: local excision or a wait-and-see policy [23–26]. In this approach, accurate selection of ypN0 patients is essential, as the nodes remain in situ. Knowledge on the effect of radiation on nodes can help a radiologist to accurately identify ypN0 patients and thus allow for an organ-preserving treatment.

### Limitations

There are some limitations to this study. First, although the data were prospectively collected as part of a study on nodal staging, it can be considered as a retrospective study. Second, the number of patients is limited. The overall number of lymph nodes, however, is large enough to allow for robust results. Third, the matching of nodes on MRI with histology may not have been 100 % accurate, and despite our efforts to match all visible nodes, this was not always possible.

## Conclusion

Our study shows that after CRT, the vast majority of the lymph nodes become smaller, and almost half of them are even no longer visible on MRI. The smaller the node, the higher the likelihood it disappears on MRI. The more a node decreases in size, the smaller the likelihood of tumoural involvement. These findings can explain the higher accuracy, particularly sensitivity, of nodal staging after CRT than in a primary staging setting. First, small nodes that are difficult to stage with morphologic criteria on MRI are no longer visible after CRT. Second, the prevalence of positive nodes is lower leading to a higher negative predictive value and thus more accurate selection of the node negative patients after CRT. This increased accuracy of nodal staging after CRT can be of clinical use in organ-preserving strategies after a good response to CRT.
